# A geometric process of evolutionary game dynamics

**DOI:** 10.1098/rsif.2023.0460

**Published:** 2023-11-29

**Authors:** Philip LaPorte, Martin A. Nowak

**Affiliations:** ^1^ Department of Mathematics, University of California, Berkeley, CA 94720, USA; ^2^ Department of Mathematics, Harvard University, Cambridge, MA 02138, USA; ^3^ Department of Organismic and Evolutionary Biology, Harvard University, Cambridge, MA 02138, USA

**Keywords:** evolutionary game theory, evolution of cooperation, direct reciprocity, Prisoner’s Dilemma

## Abstract

Many evolutionary processes occur in phenotype spaces which are continuous. It is therefore of interest to explore how selection operates in continuous spaces. One approach is adaptive dynamics, which assumes that mutants are local. Here we study a different process which also allows non-local mutants. We assume that a resident population is challenged by an invader who uses a strategy chosen from a random distribution on the space of all strategies. We study the repeated donation game of direct reciprocity. We consider reactive strategies given by two probabilities, denoting respectively the probability to cooperate after the co-player has cooperated or defected. The strategy space is the unit square. We derive analytic formulae for the stationary distribution of evolutionary dynamics and for the average cooperation rate as function of the cost-to-benefit ratio. For positive reactive strategies, we prove that cooperation is more abundant than defection if the area of the cooperative region is greater than 1/2 which is equivalent to benefit, *b*, divided by cost, *c*, exceeding $2+\sqrt{2}$. We introduce the concept of strategies that are stable with probability one. We also study an extended process and discuss other games.

## 1. Introduction

One possibility to study evolutionary dynamics in continuous strategy spaces is adaptive dynamics, which was introduced in the context of direct reciprocity [1]. The basic assumption is that mutant strategies are infinitesimally close to the resident strategy and selection follows the gradient of invasion fitness. The resulting process is a deterministic differential equation on the space of strategies [1–3]. Adaptive dynamics has been applied to many different games and questions that arise in evolutionary biology including infectious diseases [4], evolutionary dynamics with interaction structure [5], altruism in spatial models [6], evolution of genetic polymorphism [7], speciation in patch models [8], evolutionary branching [9–11], physiologically structured populations [12], the Snowdrift game [13,14], the ultimatum game [15] and memory-1 strategies of repeated games [16,17]. The theory of adaptive dynamics is developed and extended in [1,3,18–21].

Another approach to study evolutionary dynamics in continuous strategy spaces was introduced by Imhof & Nowak [22]. Here mutants are taken globally from the space of all strategies, similar to Kingman’s House of Cards model [23]. The new mutant takes over the resident with a probability that is given by the fixation probability in the frequency-dependent Moran process for a population of finite size, *N*, which is a parameter of the process. The process induces a stationary distribution on the space of all strategies which can be studied with computer simulations.

Here we consider a process of evolutionary dynamics which is similar to the Imhof–Nowak process [22], but which can be studied analytically. Mutants are taken globally from the space of all strategies. A new mutant takes over the resident based on a fitness comparison between the mutant and resident. Specifically, we study evolutionary dynamics in the space of reactive strategies for the repeated donation game. We compare our process with the adaptive dynamics of reactive strategies [1], as well as simulation results for the Imhof–Nowak process [22,24]. We also apply our process to other games, including Prisoner’s Dilemma, Stag-Hunt, Snowdrift and the continuous donation game.

Direct reciprocity is a mechanism for evolution of cooperation [25], which is based on repeated encounters between the same individuals. Cooperation can be achieved if players use conditional strategies that depend on the outcome of previous interactions [26–42]. Well-known strategies include tit-for-tat [28], generous tit-for-tat [30] and win-stay, lose-shift [31]. Partner strategies aim to share the pay-off for mutual cooperation, but are ready to fight back when being exploited. Rival strategies strive for a unilateral advantage. In general, partners but not rivals manage to achieve cooperation in direct reciprocity [39].

## 2. Methods

In the infinitely repeated donation game, two players decide simultaneously in each round whether to cooperate (*C*) or to defect (*D*). (For the asynchronous donation game or Prisoner’s Dilemma, see [43–47].) Cooperation incurs a cost *c* while conferring a benefit *b* on the other player [17,48–52]. The pay-off matrix is given by

2.1 $$\begin{array}{cc} & \begin{array}{cc} C & \;D \end{array}\\ \begin{array}{c} C\\ D \end{array} & \left(\begin{array}{cc} b-c & -c\\ b & 0 \end{array}\right) \end{array}.$$

The game is a Prisoner’s Dilemma if *b* > *c* > 0 [26]. We set *b* = 1 without loss of generality, so that the cost-to-benefit ratio is represented by *c*.

Both players can base their decision in each round on the previous move of their opponent. In particular, we consider reactive strategies [1,22,24,30,53–55]. A reactive strategy *S*(*p*, *q*) is given by two probabilities, *p*, *q*, and a choice of first move which is either *C* or *D*. The parameters *p* and *q* denote, respectively, the probability to cooperate if the opponent previously cooperated or if the opponent previously defected. The long-run behaviour of reactive strategies turns out not to depend on the choice of first move, so now we omit it.

The space of reactive strategies is given by the standard unit square, [0, 1]^2^. The four corner points of the square are the pure reactive strategies always defect, ALLD, *S*(0, 0); always cooperate, ALLC, *S*(1, 1); tit-for-tat, TFT, *S*(1, 0); and reverse tit-for-tat, RTFT, *S*(0, 1). RTFT performs poorly in evolutionary simulations [56,57]. The space of reactive strategies also includes generous tit-for-tat, GTFT, *S*(1, *x*) with 0 < *x* < 1 [30,58,59]. The interior points of the square are stochastic strategies. The centre of the square is given by the random strategy, *S*(1/2, 1/2).

A reactive strategy *S*(*p*, *q*) is determined uniquely by the two parameters

2.2 r=p−q

and

2.3 $$s=\frac{q}{1-p+q}.$$

The parameter *s* is the cooperativity of the strategy *S*(*p*, *q*), defined as the average frequency at which *S* cooperates in the long run when facing another player who also uses strategy *S*. Level sets of *s* are straight lines radiating from *S*(1, 0) (TFT).

The parameter *r* measures how much more frequently the strategy *S*(*p*, *q*) cooperates against ALLC than against ALLD. Level sets of *r* are straight lines with slope 1. We refer to the strategies with *r* > 0 as positive reactive strategies; to those with *r* < 0 as negative reactive strategies; and to those with *r* = 0 as unconditional strategies. Of the four pure reactive strategies, TFT is positive reactive, RTFT is negative reactive, and ALLD and ALLC are unconditional.

The repeated interaction between a player using strategy *S* and a co-player using strategy *S*′ is described by a Markov chain with state space {*CC*, *CD*, *DC*, *DD*} and a 4 × 4 transition matrix (see [1,60] for details). The long-run average pay-off for *S* versus *S*′ is described by an explicit formula

2.4 $$A(S,S^{\prime })=\frac{q^{\prime }+qr^{\prime }-c(q+q^{\prime }r)}{1-rr^{\prime }}.$$

The adaptive dynamics of reactive strategies are well understood [1]. The region *r* > *c* is called the cooperative region or *C*-region. Evolution into or out of the cooperative region does not occur. Inside the cooperative region *p* and *q* both increase, and outside of the cooperative region *p* and *q* both decrease. Evolution is assumed to continue on the boundary of the space. The attracting fixed points on the boundary occur at two loci: {S(p,0) | p∈[0,c]}, which consists of ALLD and some variants, and {S(1,q) | q∈(0,1−c]}, which consists of GTFT.

The Imhof–Nowak process for reactive strategies has been studied by computer simulations [22,24]. The most successful strategies are ALLD and GTFT. The simulated stationary distribution is heavily concentrated in the vicinity of those strategies. This process differs from adaptive dynamics in some respects. For example, the population can evolve out of a small neighbourhood of ALLD and into a region of high cooperativity. The population may also evolve into and out of the cooperative region.

We formulate a new discrete-time stochastic process as follows. In a homogeneous population playing strategy *S* the pay-off *A*(*S*, *S*) = (1 − *c*)*s*. For an invading strategy *S*′, the pay-off is *A*(*S*′, *S*). The invasion is favoured by selection (the invasion fitness is positive) if

2.5 $$\;A(S^{\prime },S)>A(S,S)\;⟺\;(r-c)(s^{\prime }-s)>0.$$

In this case, the invasion fitness, *A*(*S*′, *S*) − *A*(*S*, *S*) is positive.

Evolutionary dynamics begins with a homogeneous population using some reactive strategy, *S*. Then a potential invader strategy, *S*′, is taken from a uniform distribution over the whole strategy space. Hence, we admit non-local mutations [22,61]. With probability *μ* the invader replaces the resident independent of any pay-off consideration. The parameter, *μ*, adds noise or random drift. Positive *μ* ensures that the process is ergodic with a unique stationary distribution. With probability 1 − *μ* a pay-off comparison is performed. In the basic process, the pay-off comparison is as follows:

2.6 $$\{\begin{array}{c} \mathrm{if}\;A(S^{\prime },\;S)>A(S,\;S)\Rightarrow S^{\prime }\;\mathrm{replaces}\;S\;\\ \mathrm{if}\;A(S^{\prime },\;S)\leq A(S,\;S)\Rightarrow S\;\mathrm{remains}.\; \end{array}$$

The assumption is that a new strategy which achieves a lower pay-off will immediately die out, and a new strategy which achieves a higher pay-off will be quickly adopted by the rest of the population. For the extended process, the pay-off comparison is as follows:

2.7 $$\{\begin{array}{rl} & \;\mathrm{if}\;A(S^{\prime },\;S)>A(S,\;S)\;\mathrm{and}\;A(S^{\prime },\;S^{\prime })\geq A(S,\;S^{\prime })\Rightarrow S^{\prime }\;\mathrm{replaces}\;S\;\\ & \mathrm{if}\;A(S^{\prime },\;S)>A(S,\;S)\;\mathrm{and}\;A(S^{\prime },\;S^{\prime })<A(S,\;S^{\prime })\\ & \;\;\;\Rightarrow S^{\prime }\;\mathrm{replaces}\;S\;\mathrm{with}\;\mathrm{probability}\;w\;\\ & \mathrm{if}\;A(S^{\prime },\;S)\leq A(S,\;S)\Rightarrow S^{\;}\mathrm{remains}. \end{array}$$

Here the assumption is that a new strategy which achieves a lower pay-off will immediately die out; a new strategy which dominates the current strategy will take over the population; and a new strategy which stably coexists with the current strategy will take over the population with constant probability *w* and will die out with probability 1 − *w*. If *w* = 1 then the extended process reduces to the basic process. For more variants of the process, see the electronic supplementary material, appendix.

It does not matter whether the inequalities in (2.6) and (2.7) are strict or weak. With probability one, equality never occurs. As in the Imhof–Nowak process, the population is homogeneous after each round. This simplification allows us to carry out a formal analysis. However, we note that diversity in behaviour [62,63], strategy updating [64] and social status [65] in a population can play important roles in the evolution of cooperation. For other results of evolutionary game dynamics in heterogeneous populations, see [42,66–74].

**Figure 1.**
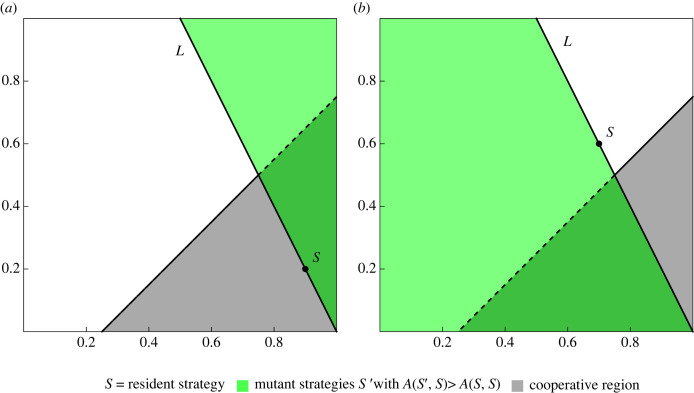
A geometric process of evolutionary dynamics. The strategy (or phenotype) space is given by the unit square. A strategy is a point in the unit square. To determine whether a resident strategy, *S*, can be invaded by a mutant strategy, *S*′, we draw a straight line, *L*, between *S* and the corner point (1, 0). (*a*) If *S* is within the grey region of the strategy space, then all strategies above *L* can invade. (*b*) If *S* is outside the grey region of the strategy space, then all strategies below *L* can invade. In both (*a*) and (*b*), the green area indicates the location of successful invaders. If *S*′ fails to invade *S*, then *S* remains resident and we generate a new *S*′. If *S*′ invades *S* it becomes the new *S*. The process is iterated many times with every new *S*′ drawn from a uniform distribution on the unit square. To this basic process we can add noise. With probability *μ* < 1 any *S*′ replaces *S*. With probability 1 − *μ* we apply the above criterion. We are interested in the stationary distribution over the strategy space that is generated by the evolutionary process. This geometric process arises when considering reactive strategies, given by *S*(*p*, *q*), in the repeated donation game. Here *p* and *q* are the probabilities to cooperate if the opponent has cooperated or defected, respectively. The grey region corresponds to the cooperative region. The line, *L*, contains all strategies that have the same cooperativity as *S*. Strategies above *L* have higher cooperativity. Strategies below *L* have lower cooperativity. If a strategy *S* is in the cooperative region, it can be invaded by all strategies that have higher cooperativity. If a strategy *S* is outside the cooperative region, it can be invaded by all strategies that have lower cooperativity. The parameter *c* determines the size of the cooperative region, shown here for *c* = 0.25.

**Figure 2.**
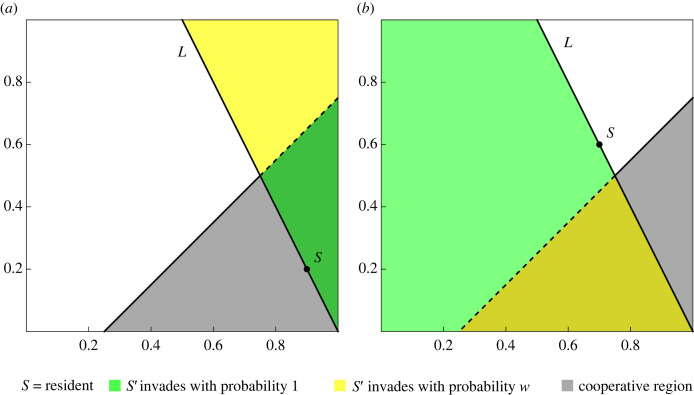
The extended process. We generalize the basic process described in figure 1. As before, the strategy (or phenotype) space is given by the unit square. A strategy is a point in the unit square. The population is assumed to be homogeneous with resident strategy *S*. In each round, a random challenger *S*′ is introduced and compared with *S* to determine whether it will replace *S*. In both (*a*) and (*b*), the green area indicates the location of invaders which always successfully replace the resident. These strategies dominate *S*. The yellow area indicates the location of invaders which replace *S* with probability *w*. These strategies have higher pay-off against the resident *S*, but lower pay-off against themselves. As the figure indicates, the green and yellow regions are determined by three pieces of data: *L*, the line connecting *S* with TFT; the cooperative region, shown in grey; and whether *S* is in the cooperative region. If *S*′ fails to invade *S*, then *S* remains resident and we generate a new *S*′. If *S*′ invades *S* it becomes the new *S*. The process is iterated many times with every new *S*′ drawn from a uniform distribution on the unit square. We add noise as we did to the basic process: with probability *μ*, the mutant replaces the resident unconditionally rather than in accordance with the relevant pay-off comparison. Parameter *c* = 0.25.

Both the basic and the extended process have simple geometric interpretations on the space of reactive strategies, see figures 1 and 2. We study both processes on the space of reactive strategies, as well as the subspace of positive reactive strategies.

We also explore other 2 × 2 games including Prisoner’s Dilemma [75], the Stag-Hunt [76,77] and the Snowdrift game [14,78–80]. We predict the peaks of the stationary distribution for our evolutionary process by studying the strategies which are ‘stable with probability 1’ or SP1. A strategy *S* is SP1 if $P(A(S^{\prime },S)<A(S,S))=1$ for a random strategy *S*′. Therefore, an SP1 strategy is robust against invasion with probability 1. Furthermore, the strategies in a small neighbourhood of an SP1 strategy are robust against invasion with high probability. We classify the SP1 reactive strategies for any 2 × 2 pay-off matrix.

## 3. Results

Formal derivations of the following results can be found in the electronic supplementary material, appendix.

### 3.1. Positive reactive strategies

The positive reactive strategies are defined by *p* > *q*. They occupy half of the square [0, 1]^2^. The entire cooperative region consists of positive reactive strategies. The cooperative region occupies a fraction *z* of the space of positive reactive strategies. It is easy to see that

3.1 $$z={\left(1-c\right)}^{2}.$$

A random positive reactive strategy has cooperativity 1/2 on average, which is the same as a random reactive strategy.

#### 3.1.1. The basic process

In the basic process (2.6), the mutant *S*′ replaces the resident *S* if *A*(*S*′, *S*) > *A*(*S*, *S*). The unique stationary distribution on positive reactive strategies has a piecewise formula

3.2 $$F(p,q)=\frac{2(1+\mu )}{\mu^{-z}+\mu^{z-1}}\cdot \left\{ \begin{array}{c} \;{\left(s+\mu (1-s)\right)}^{z-1}{\left(1-(1-\mu )s\right)}^{-1-z}\;\;r>c\\ \;{\left(s+\mu (1-s)\right)}^{z-2}{\left(1-(1-\mu )s\right)}^{-z}\;\;r<c. \end{array}\right.$$

**Figure 3.**
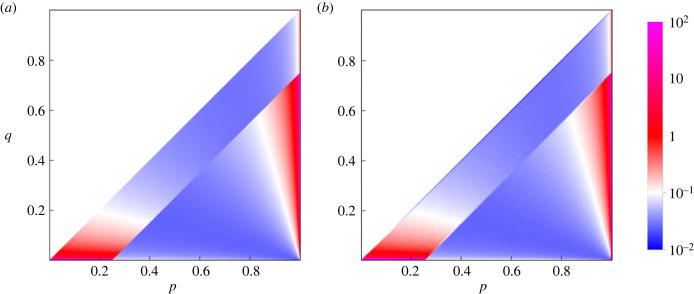
Stationary distribution on the space of positive reactive strategies, which are given by *p* − *q* > 0. (*a*) The stationary distribution for the basic process according to (3.2). (*b*) The distribution obtained by numerical simulation. Parameter values: *c* = 0.25 and *μ* = 10^−4^.

Recall the parameters *r* and *s* are given by *r* = *p* − *q* and *s* = *q*/(1 − *r*). Figure 3 gives a density plot of the stationary distribution. The stationary average cooperativity *C* is defined by integrating *s* over the stationary distribution (3.2):

3.3 C=∫sF(p,q) dp dq.

An analytic formula for *C* is given by

3.4 $$C=\frac{\alpha +\beta (2z-1)(1+\mu )}{z{\left(1-\mu \right)}^{2}(1+\mu^{2z-1})}.$$

We have used the notation

$$\alpha =1+\mu +\mu^{2z}(\mu (2z-1)-1)+z(\mu (\mu -2)-1)$$

and

$$\beta ={\;}_{2}F_{1}(1,-z,1-z,-\mu )+\frac{z}{1+z}(\mu^{z}\Gamma (2+z)\Gamma (-z)-\mu^{1+2z}{\;}_{2}F_{1}(1,1+z,2+z,-\mu )).$$

Here _2_*F*_1_ is the Gaussian hypergeometric function, and Γ is the gamma function. The *α* and *β* terms in (3.4) have comparable magnitude for most *z*.

As *μ* varies, formula (3.4) has a stationary point *z* = 1/2, *C* = 1/2. In other words, regardless of the level of noise, cooperation and defection are equally abundant on average if the cooperative region occupies half of the strategy space. This happens when the cost-to-benefit ratio $c=1-\sqrt{z}=1-1/\sqrt{2}\approx 0.293$.

In the limit *μ* → 0 of vanishing noise, *C* converges to 0 or 1, depending on the cost-to-benefit ratio. We have

3.5 $$\lim_{\mu \to 0}C=\left\{ \begin{array}{c} \;0\;\;z<1/2\\ \;1\;\;z>1/2. \end{array}\right.$$

**Figure 4.**
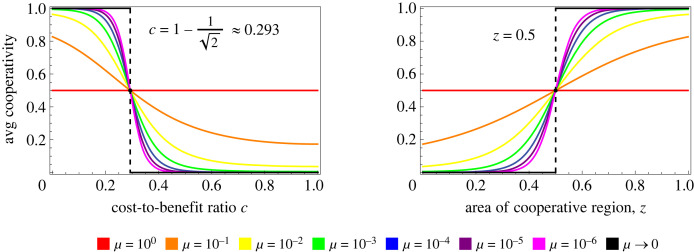
Average cooperation rate in the stationary distribution for positive reactive strategies. Here we plot the stationary average cooperativity for our process on positive reactive strategies—those strategies which are more likely to cooperate if the opponent cooperated in the previous round. Stationary average cooperativity is a function (3.4) of the cost-to-benefit ratio *c*, or, alternatively, the normalized area *z* = (1 − *c*)^2^ of the cooperative region. It also depends on the noise parameter *μ*. When *μ* = 1, our process simply selects a random strategy uniformly in each round. When *μ* = 0, only mutants with higher invasion fitness can replace a resident. If *μ* = 0, the process is no longer mixing and a unique stationary distribution does not exist: moreover, the *L*^1^ limit of the stationary distribution as *μ* → 0 does not exist, so we cannot compute its average cooperativity. However, if we interchange the order of the limit and the cooperativity function, we get a perfectly coherent result. The limit lim_*μ*→0_C is a step function shown in both panels of figure and described in (3.5). Notice that independently of *μ*, cooperativity is greater than 1/2 if and only if $c<1-1/\sqrt{2}$. Geometrically, this is precisely the value of *c* that corresponds to a cooperative region of area 1/4 (*z* = 1/2 after normalizing by the positive reactive strategies), shown in figure 7.

In other words, the population will sustain full cooperation on average if *z* > 1/2 and full defection on average if *z* < 1/2. The detailed relationship between *C*, *z* and *μ* is illustrated in figure 4. In this figure we observe the stationary point *z* = 1/2, *C* = 1/2 and the limit lim_*μ*→0_*C*.

#### 3.1.2. The extended process

The basic process is simple because mutants which have higher invasion fitness always take over the population—even mutants which are themselves vulnerable to invasion by the replaced strategy. In the extended process (2.7), we introduce a fixed probability *w* to accept these ambivalent mutants. More precisely, we specify that a mutant *S*′ replaces a resident *S* if *A*(*S*′, *S*) > *A*(*S*, *S*) and *A*(*S*′, *S*′) ≥ *A*(*S*, *S*′); or—with a fixed probability *w*—if *A*(*S*′, *S*) > *A*(*S*, *S*) and *A*(*S*′, *S*′) < *A*(*S*, *S*′). The idea is that even if *S* and *S*′ can each invade each other, random drift prevents their coexistence in the long term. In this case, the mutant is assumed to take over the population with probability *w*. The extended process coincides with the basic process when *w* = 1. A simple geometric interpretation of the extended process is illustrated in figure 2.

As the parameters *μ* ∈ (0, 1) and *w* ∈ [0, 1] vary, we have the same stationary point as for the basic process: *z* = 1/2, *C* = 1/2. Cooperation and defection are equally abundant on average precisely when the cooperative region occupies half of the strategy space.

### 3.2. Stationary distribution for reactive strategies

We also find the stationary distribution for the basic process on all reactive strategies. The unique stationary distribution on [0, 1]^2^, before normalization, is given piecewise by

3.6 $$F(p,q)=\left\{ \begin{array}{cc} \;\exp\left(\int_{0}^{s}b(\sigma )\;d\sigma \right) & r>c\\ \frac{1-a(s)(1-\mu )}{\mu +a(s)(1-\mu )}\exp(\int_{0}^{s}b(\sigma )\;d\sigma ) & r<c.\; \end{array}\right.$$

We use the notation

3.7 $$b(s)=\frac{\left(1-\mu \right)}{2}\left(\frac{z+2a^{\prime }(s)}{1-a(s)(1-\mu )}-\frac{1-z-2t(s)+t{\left(s\right)}^{2}}{\mu +a(s)(1-\mu )}\right)$$

and

3.8 $$a(s)=\{\begin{array}{cc} \frac{s}{2(1-s)}\; & s<\frac{1}{2}\;\\ \frac{3}{2}-\frac{1}{2s}\; & s>\frac{1}{2}\; \end{array}\;\;\;t(s)=\{\begin{array}{cc} \frac{s}{\left(s-1\right)}\; & s<\frac{1}{2}\;\\ \frac{\left(s-1\right)}{s}\; & s>\frac{1}{2}.\; \end{array}$$

**Figure 5.**
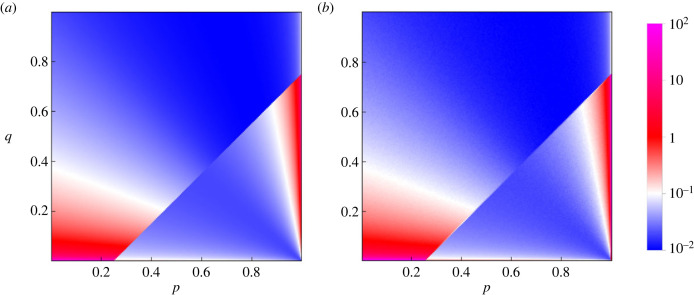
Stationary distribution on the space of all reactive strategies. (*a*) The stationary distribution for the basic process according to (3.6). (*b*) The distribution obtained by numerical simulation. Parameter values: *c* = 0.25 and *μ* = 10^−4^.

Figure 5 gives a density plot. The plot resembles simulations of the Imhof–Nowak process [22,24]. However, we derive more precise results using our formula. The local maxima of the stationary distribution occur at the loci {*S*(*p*, 0), *p* ∈ [0, *c*)} and {*S*(1, *q*), *q* ∈ (0, 1 − *c*)}. As mentioned in Methods, these are precisely the attracting fixed points on the boundary for adaptive dynamics. The first locus consists of ALLD together with some variants of zero cooperativity, and the second locus consists of versions of GTFT. The global maximum is GTFT when

3.9 $$\int_{0}^{1}b(\sigma )\;d\sigma <|\ln\mu |.$$

This is a useful linear inequality in the variable *z* = (1 − *c*)^2^. For instance, at *μ* = 10^−4^, we calculate that GTFT is the most successful strategy whenever *c* < 0.224. This is similar to the empirical result *c* < 1/4 for the Imhof–Nowak process [24].

**Figure 6.**
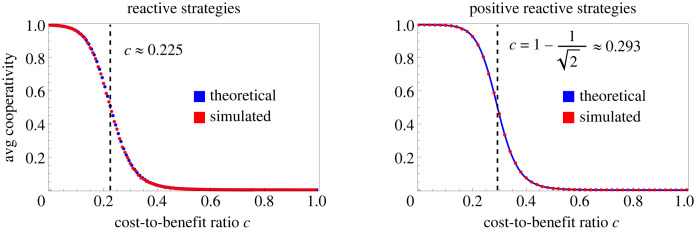
Average cooperation rate in the stationary distribution for reactive and positive reactive strategies. Here we plot the stationary average cooperativity for our process on the space of reactive strategies and the subspace of positive reactive strategies, respectively. The positive reactive strategies are those strategies which are more likely to cooperate if the opponent cooperated in the previous round. For positive reactive strategies, shown on the right, the stationary distribution (3.2) has average cooperativity given by (3.4) as a function of the cost-to-benefit ratio *c* and the noise parameter *μ*. We fix *μ* = 10^−4^ and plot the curve (3.4) in blue along with a number of points in red obtained from simulation. The blue and red data give perfect agreement, confirming the accuracy of the simulation. The value $c=1-1/\sqrt{2}$, described by a dashed vertical line, corresponds to an average cooperativity of 1/2, independently of *μ*. Geometrically, this is precisely the value of *c* that corresponds to a cooperative region of relative size 1/2, shown in figure 7. On the left, we show the equivalent figure for the process on all reactive strategies. In this case, we do not have a convenient formula such as (3.4), so we plot theoretical data numerically in blue and compare with simulation in red. Here we achieve cooperativity 1/2 at *c* ≈ 0.225. The corresponding cooperative region is shown in figure 7.

**Figure 7.**
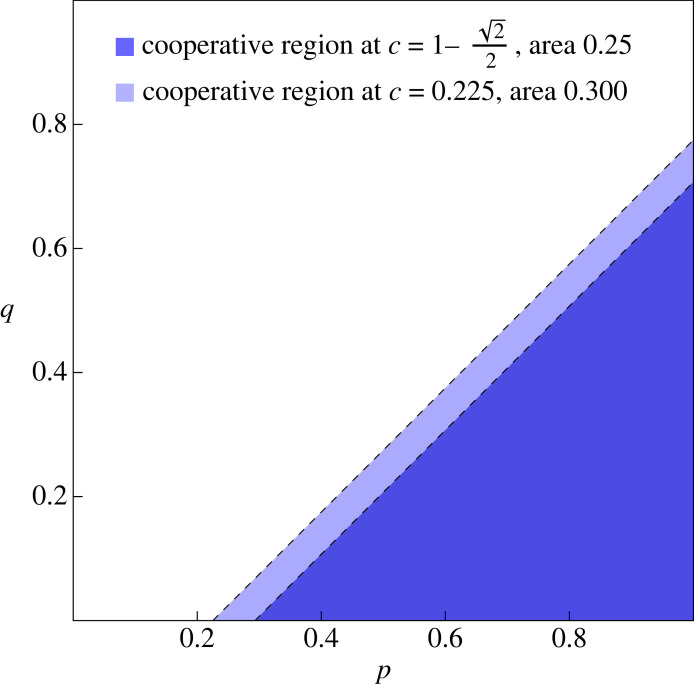
Cooperative regions for the critical *c* values for reactive and positive reactive strategies. For these cooperative regions the average cooperation in the stationary distribution is 1/2. For positive reactive strategies, this occurs at $c=1-1/\sqrt{2}$, corresponding to a cooperative region of relative size 1/2: equal to its complement. For reactive strategies, this occurs at *c* ≈ 0.225, corresponding to a cooperative region about 20% larger. In other words, the cost of allowing negative reactive strategies as mutants is a 20% increase in the size of the cooperative region required to achieve cooperativity greater than 1/2.

**Figure 8.**
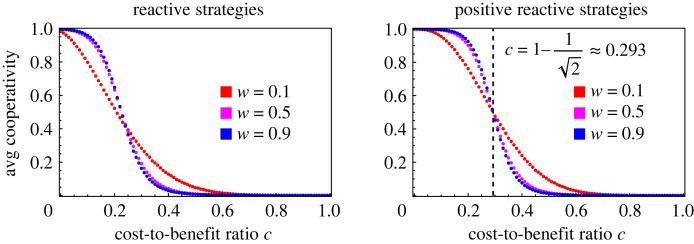
Average cooperation rate for the extended process on reactive and positive reactive strategies. Here we plot the stationary average cooperativity for the extended process on the space of reactive strategies and the subspace of positive reactive strategies, respectively, based on simulation data. The positive reactive strategies are those strategies which are more likely to cooperate if the opponent cooperated in the previous round. For positive reactive strategies, shown on the right, the value $c=1-1/\sqrt{2}$—described by a dashed vertical line—corresponds to an average cooperativity of 1/2. This is true regardless of *μ* and *w*. Geometrically, this is precisely the value of *c* that corresponds to a cooperative region of relative size 1/2, shown in figure 7. On the left, we show the equivalent figure for the process on all reactive strategies. Parameter *μ* = 10^−4^.

We can also calculate when cooperation is more abundant on average than defection in evolutionary dynamics. At *μ* = 10^−4^, we find numerically that *C* > 1/2 when *c* < 0.225. This is slightly more restrictive than the condition $c<1-1/\sqrt{2}\approx 0.293$ for positive reactive strategies. The comparison is illustrated in figures 6 and 7. Note that the results for reactive strategies are numerically quite similar to those for positive reactive strategies in other respects (cf. figures 5 and 3), including for the extended process (figure 8).

### 3.3. Other games

We apply our process to other 2 × 2 games with a general pay-off matrix

3.10 $$\left(\begin{array}{cc} R & S\\ T & P \end{array}\right).$$

The basic process and the extended process are invariant under transformations

3.11 $$\left(\begin{array}{cc} R & S\\ T & P \end{array}\right)\to \left(\begin{array}{cc} \gamma R+\delta & \gamma S+\delta \\ \gamma T+\delta & \gamma P+\delta \end{array}\right),\;\gamma >0.$$

We propose the concept of a ‘stable with probability 1’ or SP1 strategy to indicate a strategy *S* which is stable against invasion with probability 1. Precisely, we say that *S* is an SP1 strategy if for a random strategy *S*′, we have $P(A(S^{\prime },S)<A(S,S))=1$. The SP1 strategies for an arbitrary 2 × 2 game with pay-off matrix (3.10) are classified by the following:

3.12 $$\text{if}\;R+P-S-T=0\Rightarrow \text{SP1\textbackslash{} strategies\textbackslash{} are}\;A_{1}\cup A_{2}$$

and

3.13 $$\begin{array}{r} \text{if}\;R+P-S-T\neq 0\Rightarrow \text{SP1\textbackslash{} strategies\textbackslash{} are}\;A_{1}^{\prime }\cup A_{2}^{\prime }\cup A_{3}^{\prime }\setminus B. \end{array}$$

The sets *A*_1_, *A*_2_, *A*_1_′, *A*_2_′, *A*_3_′, *B* are defined by

3.14 $$A_{1}=\{S(p,0)\;|\;p(T-P)<P-S\},$$

3.15 $$A_{2}=\{S(1,q)\;|\;(1-q)(S-R)<R-T\},$$

3.16 $$A_{1}^{\prime }=\{S(p,0)\;|\;p\max(T-P,R-S)\leq P-S\},$$

3.17 $$A_{2}^{\prime }=\{S(1,q)\;|\;(1-q)\max(S-R,P-T)\leq R-T\},$$

3.18 $$\begin{array}{rl} A_{3}^{\prime }=\{ & S(p,q)\;|\;(R+P-S-T)r<0\;\text{and}\\ & \;q=\frac{1-r}{1+r}\frac{P-S+(P-T)r}{R+P-S-T}\} \end{array}$$

3.19 andB={S(1,1) | R=T}∪{S(0,0) | P=S}.

An interpretation of these sets is as follows. If *R* + *P* − *S* − *T* = 0, then the set *A*_1_ contains the strategies of cooperativity 0 which are attractive under adaptive dynamics. It is a subinterval of the set *q* = 0. The set *A*_2_ contains the strategies of cooperativity 1 which are attractive under adaptive dynamics. It is a subinterval of the set *p* = 1.

If *R* + *P* − *S* − *T* ≠ 0, then the set $A_{1}^{\prime }\setminus B$ contains the strategies of cooperativity 0 which are attractive under adaptive dynamics and which ALLC cannot invade. This is a subinterval of the set *q* = 0. The set $A_{2}^{\prime }\setminus B$ contains the strategies of cooperativity 1 which are attractive under adaptive dynamics and which ALLD cannot invade. This is a subinterval of the set *p* = 1. The set *A*′_3_ consists of the attracting fixed points of adaptive dynamics which have cooperativity strictly between 0 and 1. This is a smooth curve restricted to either the positive reactive or negative reactive strategies.

**Figure 9.**
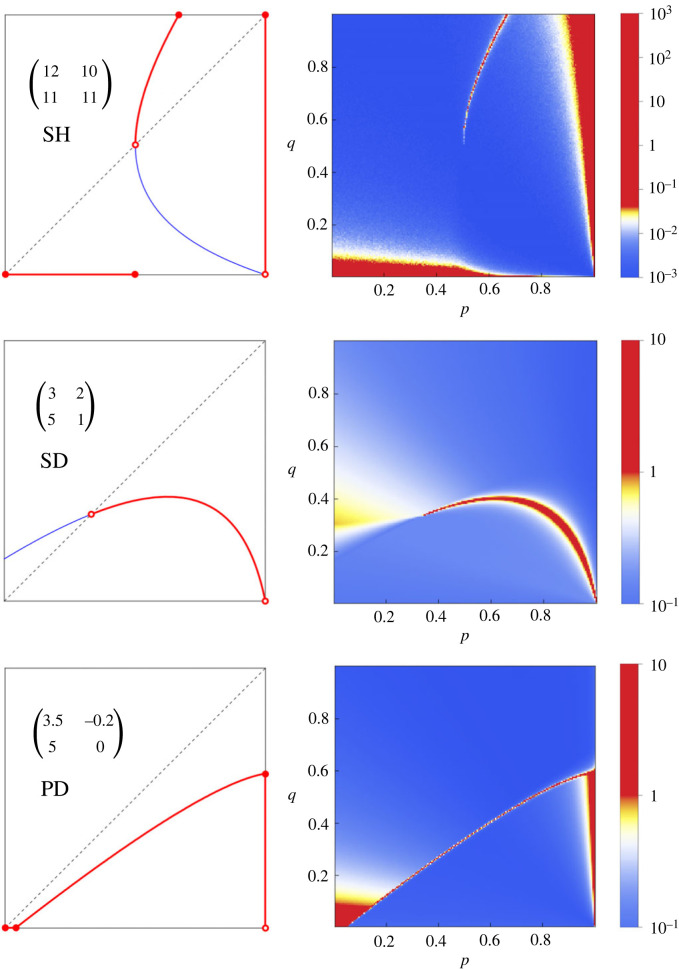
The process for other 2 × 2 games. We simulate the process for other 2 × 2 games besides the donation game. We have chosen examples of Stag-Hunt (SH), characterized by *R* > *T* = *P* > *S*; Snowdrift (SD), characterized by *T* > *R* > *S* > *P*; and Prisoner’s Dilemma (PD) with *T* > *R* > *P* > *S* and 2*R* > *T* + *S*. Each row of the figure represents one of these games. The left panel of each row is a diagram showing the location of the ‘stable with probability 1’ (SP1) strategies in red. These strategies are robust with probability 1 against invasion by a random mutant. SP1 strategies potentially come in three types: those with *p* = 1, those with *q* = 0, and those on the boundary of the cooperative region for that game [1]. The boundary of the cooperative region may cut across the line *p* = *q*. In that case, the unique intersection point is an equalizer (pay-off against it is constant). The rest of the boundary is divided into two halves. The portion on the side (*R* + *P* − *S* − *T*)(*q* − *p*) > 0 consists of SP1 strategies. The portion on the other side consists of strategies which can be invaded by a random strategy with probability 1. These are shown in blue. In the right panel of each row we show the simulated stationary distribution. Red and blue signify areas of high and low probability density, respectively. Each distribution is concentrated near the SP1 strategies. We have set the noise parameter *μ* = 10^−4^.

We simulate the basic process for a few important games. These include Stag-Hunt [76], defined by *R* > *T* ≥ *P* > *S*; Snowdrift, defined by *T* > *R* > *S* > *P* [14]; and Prisoner’s Dilemma [75], defined by *T* > *R* > *P* > *S* and 2*R* > *S* + *T*. In each case we illustrate the set of SP1 strategies and the stationary distributions which arise from the basic process (figure 9). We observe that the high-density areas of the stationary distribution are localized strikingly around the SP1 strategies.

### 3.4. The continuous donation game

We establish a relationship between two versions of the repeated donation game. First, note that the space of all possible deterministic strategies in the repeated donation game is discrete:

(a) in each round, there are two discrete actions *C* and *D*
(b) the chosen action in each round is completely determined by the finite history of play.

There are two methods of producing continuous strategy spaces. The first method relaxes condition (b) by allowing probabilistic strategies. Examples include the reactive strategies *S*(*p*, *q*) which we have studied.

The second method relaxes condition (a) by allowing a continuum of actions in every round, interpolating between *C* and *D*. The result is called the continuous donation game [32,81–83].

In the continuous donation game, players choose a degree of cooperation *λ* ∈ [0, 1] in each round. This entails paying a cost *λc* to give the other player a benefit *λb*. As usual we assume *b* = 1 > *c* > 0. A reactive strategy in the repeated continuous donation game is a function *λ* : [0, 1] → [0, 1] which specifies a degree of cooperation based on the opponent’s degree of cooperation in the previous round. In general, *λ* may be arbitrary; however, if *λ* is a linear function [81,82], then it can be expressed uniquely in the form

3.20 λ(x)=px+q(1−x).

Note that *p* = *λ*(1) and *q* = *λ*(0). We call such a strategy *T*(*p*, *q*). Two linear reactive strategies *T*, *T*′ give rise to a unique stable equilibrium, with pay-off *A*(*T*, *T*′).

Our notation suggests a correspondence *S*(*p*, *q*) ↔ *T*(*p*, *q*) between (stochastic) reactive strategies in the repeated donation game, and linear reactive strategies in the repeated continuous donation game. In fact, this correspondence is an equivalence of pay-offs:

3.21 $$A(S,S^{\prime })=A(T,T^{\prime }).$$

Therefore, our results also hold for linear reactive strategies in the continuous donation game. The positive reactive strategies correspond to linear reactive strategies with positive slope.

## 4. Conclusion

We have studied a geometric process of evolutionary game dynamics on the space of reactive strategies. Reactive strategies are given by two parameters, *p* and *q*, which denote, respectively, the probabilities to cooperate after the opponent has cooperated or defected. The space of reactive strategies is given by the unit square, [0, 1]^2^. Positive reactive strategies are those reactive strategies for which *p* > *q*. Our evolutionary process describes transitions from one homogeneous state to another. A resident strategy is challenged by an invader. In the basic process, the invader is rejected if the invasion fitness is negative, the invader is adopted if the invasion fitness is positive. In the extended process, the invader is rejected if the invasion fitness is negative, the invader is accepted if it dominates the resident, or the invader is accepted with probability *w* if resident and invader can mutually invade each other. In both cases we add noise: with probability *μ* any invader is accepted regardless of pay-off considerations.

For the basic process, we derive an analytic formula for the stationary distribution on the strategy space. For positive reactive strategies, we derive an analytic formula for the average cooperation rate as a function of the cost-to-benefit ratio. For positive reactive strategies, we also prove that the average cooperation rate exceeds 1/2 if the fraction of the cooperative region *z* = (1 − *c*)^2^ > 1/2, where *c* is the cost-to-benefit ratio. This is true for any value of the noise parameter *μ* ∈ (0, 1).

For the extended process, we prove the equivalent result: for positive reactive strategies, the average cooperation rate exceeds 1/2 if *z* = (1 − *c*)^2^ > 1/2, independently of *μ* ∈ (0, 1) and *w* ∈ [0, 1].

Our results carry over to the continuous donation game with linear reactive strategies. We have also used our evolutionary process to study other games, such as Prisoner’s Dilemma, Stag-Hunt and Snowdrift. For those games, we illustrate how the SP1 strategies correspond to peaks in the stationary distribution induced by mutation and selection.

## Data Availability

Supplementary material is available online [84].
